# Dexmedetomidine inhibits inflammatory reaction in the hippocampus of septic rats by suppressing NF-κB pathway

**DOI:** 10.1371/journal.pone.0196897

**Published:** 2018-05-03

**Authors:** Xiaobao Zhang, Fang Yan, Jiying Feng, Haitao Qian, Zhi Cheng, Qianqian Yang, Yong Wu, Zhibin Zhao, Aimin Li, Hang Xiao

**Affiliations:** 1 Department of Anesthesiology, Lianyungang Clinical College of Nanjing Medical University, Lianyungang, China; 2 Department of Basic Medical Science, Kangda College of Nanjing Medical University, Lianyungang, China; 3 Department of Toxicology, School of Public Health, Nanjing Medical University, Nanjing, China; Massachusetts General Hospital, UNITED STATES

## Abstract

Dexmedetomidine (DEX) is known to provide neuroprotective effect in the central nervous system. However, the detailed mechanism remains far more elusive. This study was designed to investigate the relevant mechanisms of DEX's neuroprotective effect. Sprague–Dawley (SD) rats were injected with dexmedetomidine and/or Lipopolysaccharide (LPS) intraperitoneally, and inflammatory cytokines in serum and in the hippocampus were measured by enzyme linked immunosorbent assay (ELISA). NF-κB in the brain tissue extracts was analyzed with western-blot. Then, we investigated whether NF-κB inhibitor prevents the elevation of inflammatory cytokines in rats injected with LPS. Our results indicated that compared with the control group, the rats exposed to LPS showed significant cognitive dysfunction. When compared to controls, the levels of TNF-α and IL-6 in the serum and hippocampus homogenate were increased in rats treated with LPS. DEX pretreatment inhibited the rats' TNF-α, IL-6 and NF-κB levels induced by LPS. In response to LPS, PDTC pretreatment restrains the production of proinflammatory cytokines (TNF-α and IL-6). Rats treated with PDTC and DEX alongside LPS exhibited less TNF-α and IL-6 than the LPS treated group. In combination, PDTC and DEX showed addictive effects. Our data suggest that DEX exerts a neuroprotective effect through NF-κB in part after LPS-induced cognitive dysfunction.

## Introduction

Neuroinflammation plays a pivotal role in the pathophysiology of neurocognitive disorders such as Alzheimer’s disease and postoperative cognitive dysfunction(POCD) [1]. Lipopolysaccharide (LPS) is one of the most potent activators for stimulating pro-inflammatory cytokines release in experimental animals and humans [2]. Studies have shown that systemic LPS causes memory impairment [3], chronic neuroinflammation, and progressive neurodegeneration [4].

As resident immune cells in the brain, microglia play an important role in various neurodegenerative diseases. Resident microglia exert protective and restorative responses to inflammation in the CNS [5]. Microglia can be activated by numerous stimulators, which exert a high expression of brain pro-inflammatory factors (i.e., TNFα, MCP-1, IL-1β, and NF-κB p65) [4]. Therefore, inhibition of the excessive microglial pro-inflammatory response may alleviate the symptom of neurodegenerative diseases [6, 7].

Dexmedetomidine (DEX) is a selective agonist of the α2-adrenoreceptor. It shows excellent sedation and analgesia during anesthesia [8]. It has been shown that DEX exerts neuroprotective effects in various animal models [9, 10]. Notably, DEX also possesses a potent anti-inflammatory effect in many cell types through inhibition of many cytokines and chemokines, including TNF-α, IL-6, and monocyte chemotactic protein-1 (MCP-1) [11, 12]. However, the cellular and molecular mechanisms that trigger the anti-inflammatory effect of DEX are still diffuse. Studies showed that LPS could stimulate proinflammatory cascades such as toll-like receptor 4 (TLR4) and NF-κB through plasma membrane, and the activation of NF-κB leads to the induction of genes encoding proinflammatory cytokines such as IL-6 and TNF-α [13, 14]. The present study aims to explore the relevant mechanisms involved in the neuroprotective effect of DEX.

## Materials and methods

### Animals and drug administration

Sprague-Dawley rats (300–350 g) were obtained from the Nanjing Medical University Animal Center. All procedures were strictly performed in accordance with the regulations of the ethics committee of the International Association for the Study of Pain and the Guide for the Care and Use of Laboratory Animals (The Ministry of Science and Technology of China, 2006). All animal research was approved by the Nanjing Medical University Animal Care and Use Committee. The rats were allowed free access to water and food. All experiments in our research were performed in accordance with the regulations of the ethics committee of the International Association for the Study of Pain and the Guide for the Care and Use of Laboratory Animals (The Ministry of Science and Technology of China, 2006). LPS (Sigma, St Louis, MO, USA) and dexmedetomidine (Hengrui, Lianyungang, China) were diluted in saline and injected intraperitoneally (IP). Controls were injected with saline. Dexmedetomidine was injected before LPS administration.

### Experimental design

#### Experiment 1

Thirty-five rats were randomly divided into seven groups (n = 5): group C (rats were intraperitoneally injected with isotonic saline); group LPS (rats were intraperitoneally injected with different concentrations of LPS: 10, 100, 1000 μg/kg); and group DEX (rats were intraperitoneally injected with different concentration of dexmedetomidine: 5, 50, 500 μg/kg). At 24 h after the injection, a Y maze test was performed to evaluate the learning ability of the rats.

#### Experiment 2

Forty rats were randomly divided into four groups (n = 10). For each group of experiments, the animals were matched by age and body weight. The groups were (1) control group; (2) LPS group; (3) different concentration dexmedetomidine group, where rats were pretreated with various concentrations of DEX (5 and 50 μg/kg) for 1 h and then injected IP with LPS (100 μg/kg). At 24 h after the behavioral test, the blood samples of all the rats were obtained from the tail veins. Then, the hippocampus was collected either for Enzyme-Linked Immunosorbent Assay (ELISA) or for Western blotting analysis.

#### Experiment 3

Forty rats were randomly divided into four groups (n = 10): group LPS (rats were intraperitoneally injected with LPS 100 μg/kg); group DEX+LPS (rats were intraperitoneally injected with dexmedetomidine 30 min before LPS injection); group PDTC+LPS (rats were intraperitoneally injected with PDTC 30 min before LPS injection); group PDTC+DEX+LPS (rats were intraperitoneally injected with PDTC 30 min before DEX injection, followed by LPS injection). The behavioral test was performed 24 h after LPS injection. Then, the hippocampus was collected for ELISA analysis.

### Behavioral analysis

The Y maze consisted of three arms with each arm at a 120° angle. Two arms featured electrical foot stimulation, whereas one arm was a safe region with illumination. Every arm had a lamp at the tail end. Each rat was placed at the end of one arm randomly and allowed to move in the maze freely. Behavioral testing of the rat was performed during the light cycle (between 9:00 a.m. and 5:00 p.m.). The test was started for 3 minutes without disturbance, and the safe region served as the new starting area. The stimulation and safe regions were randomly changed. If the rat reached the safe region within 10 s, it was considered to have learned. The learning criterion was defined as the rat reached 9 correct in 10 consecutive foot stimulations. The learning ability was recorded as the number of stimulations to reach the learning criterion. After the behavioral test, rats were anesthetized with inhalation of sevoflurane (a concentration of 3% for induction and 1% for maintenance) by a nose mask before sacrifice. The serum of rats was collected immediately, and the brains were removed quickly. The hippocampus was carefully separated from the brains and stored at −80°C until use.

### Level of TNF-α and IL-6 in serum and hippocampus

Enzyme-Linked Immunosorbent Assays were used to measure the levels of TNF-α and IL-6 in serum and hippocampus homogenate. A glass homogenizer was used to make hippocampal homogenate in saline solution in an ice bath. After centrifugation of the tissue homogenates, supernatants were collected and immediately stored at -80°C. Levels of TNF-α and IL-6 in serum and supernatants were measured with commercial ELISA kits (R&D Systems, Minneapolis, MN, USA), according to the manufacturer’s instructions.

### Western blotting

The hippocampus of the rat was homogenized in RIPA buffer. After the homogenates were centrifuged, the concentration of the hippocampus homogenate protein in each supernatant was measured with a BCA protein assay kit. After the proteins were loaded for electrophoresis, the protein gels were transferred to a polyvinylidene fluoride microporous membrane (PVDF: Millipore, Bedford, MA). The membrane was then blocked with TBS (5% skim milk) containing 0.1% Tween-20 for 1 h at room temperature. The membrane containing the transferred proteins was incubated overnight with rabbit anti-NF-κBP65 antibody (1:200; Cell Signaling, Beverly, MA) and anti-β-actin antibody (1:1000; Sigma) at 4°C. Horseradish peroxidase conjugated secondary antibodies (1:5000; Jackson ImmunoResearch, West Grove, PA) were used to detect immunoreactivity of the protein. The density of the protein band was determined using an image analysis system (Image-Pro Plus version 6.0, Media Cybernetics, Silver Spring, MD, USA).

### Statistical analysis

Results were represented as the mean ± SEM and performed in triplicate. The statistical analyses were performed using ANOVA with Graphpad Prism 5 software (version 5.01, GraphPad Software, San Diego, CA, USA), A Newman-Keuls Multiple Comparison Test was used for post hoc analysis with significance determined at a value of P < 0.05.

## Results

### Dexmedetomidine improve LPS-induced cognitive impairment

**Fig 1:**
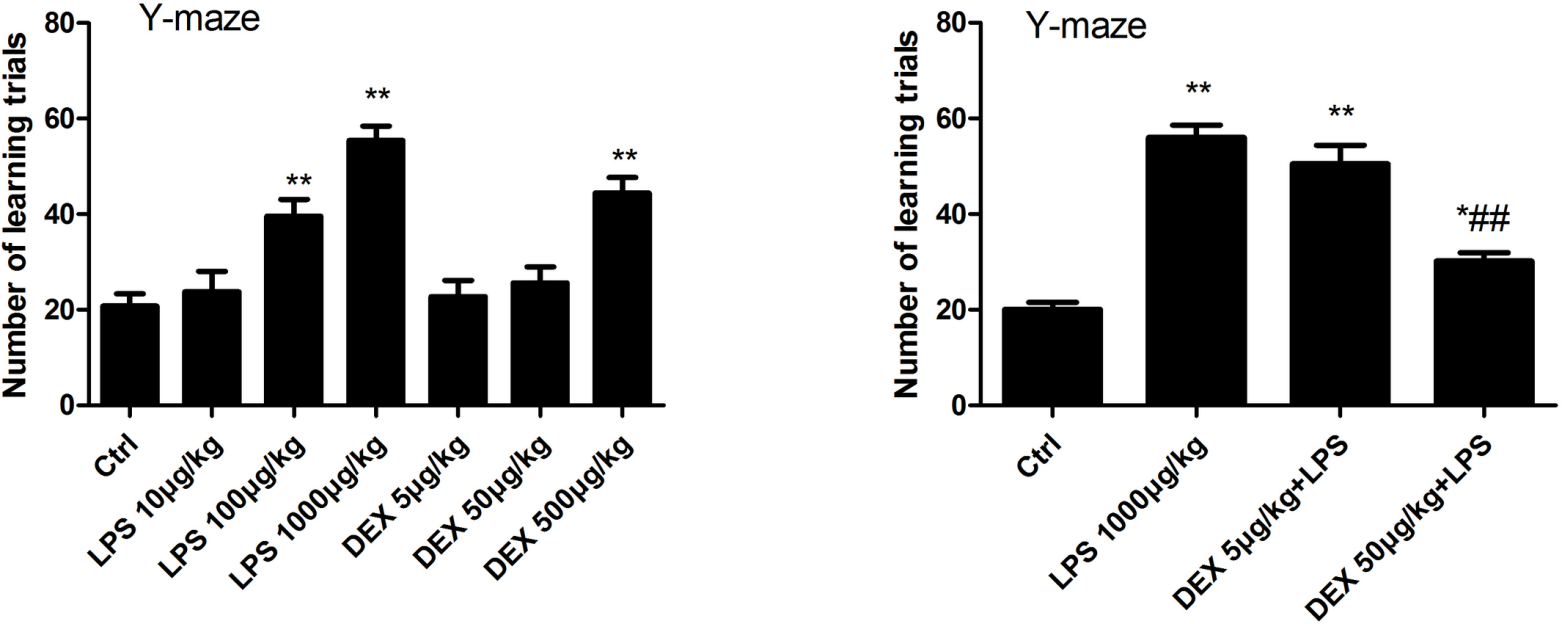
DEX improved LPS induced memory impairment. The Y-maze test was performed to observe the cognitive function of rats. (A) Rats were injected with DEX 1h prior to LPS injection, Then Y-maze test was performed to assess the rats' memory function 1d after LPS-injection. LPS significantly impaired the cognitive function of rats, DEX at concentration of 5 and 50μg/kg alone did not influence cognitive function of rats. (B) DEX at concentration of 50 μg/kg prior to LPS hindered the cognitive dysfunction, however, DEX at concentration of 500μg/kg alone significantly impaired cognitive function.

To evaluate the effect of DEX on hippocampus, rats were pretreated with DEX before stimulation. Rats were injected with DEX 1h prior to LPS injection, Then the Y-maze test was performed to assess the rats' memory function 1 d after LPS- injection. As shown in Fig 1, LPS significantly impaired the cognitive function of rats, while 5μg/kg DEX did not show cognitive protection compared to LPS group. DEX at concentration of 50 μg/kg prior to LPS hindered the cognitive dysfunction, however, DEX at concentration of 500μg/kg alone significantly impaired cognitive function. (Fig 1)(Table A and B in S1 File).

### Effect of DEX on TNF-α and IL-6 expression in rats

**Fig 2:**
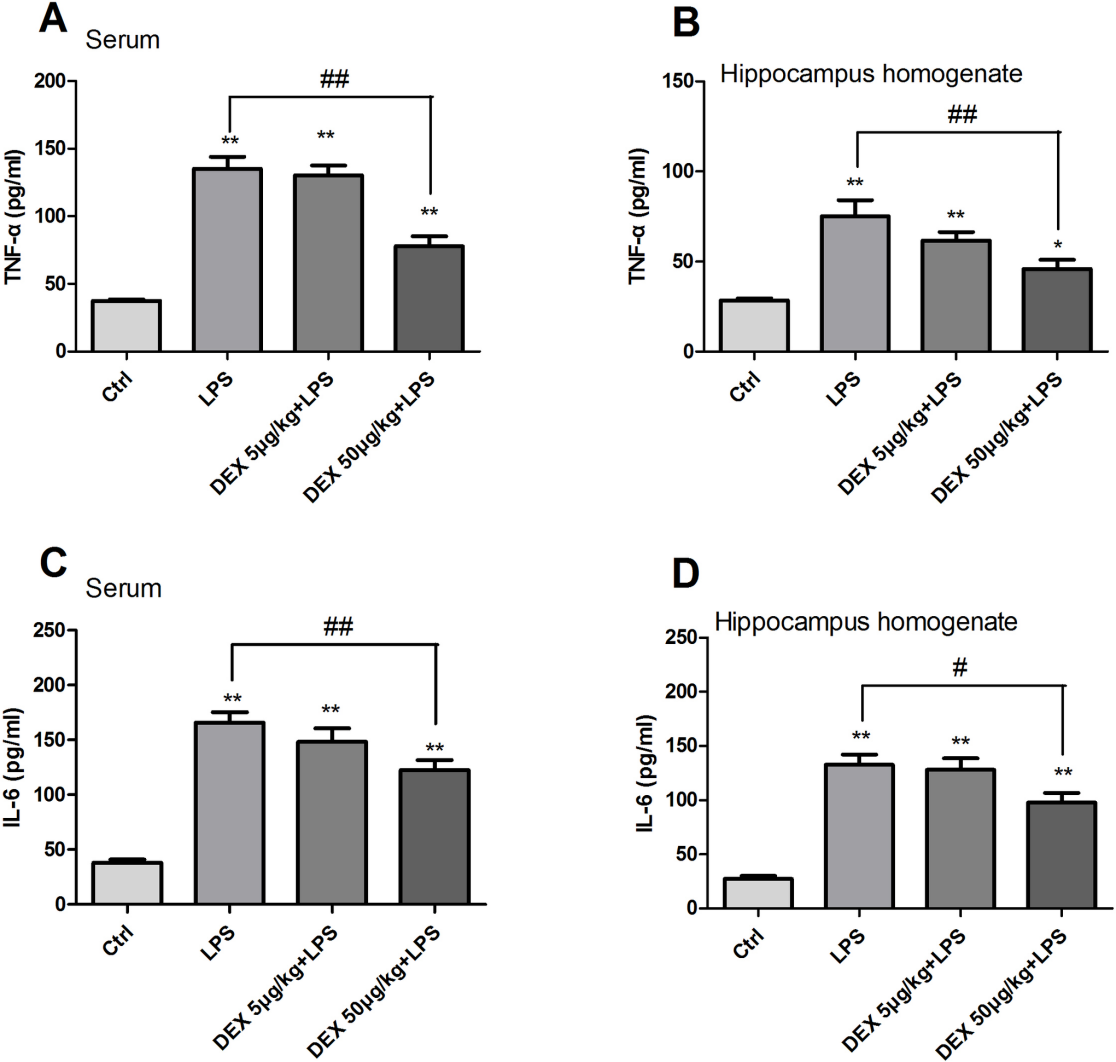
LPS induced TNF-α and IL-6 increased in rats' serum and hippocampus homogenate. Rats pretreated with DEX showed a decrease in TNF-α and IL-6 level. (A,B,C,D) ELISA showed that high dose of DEX reduced TNF-α and IL-6 protein levels in serum and hippocampus homogenate. (**P<0.01 versus control group, ^##^P<0.01 and ^#^P<0.05 versus control group; data represent the mean ± SEM for at least three separate experiments).

Cognitive impairment is connected with neuroinflammation. To estimate the effect of DEX on inflammatory factors, the levels of TNF-α and IL-6 in the serum and hippocampus homogenate were detected by ELISA. Compared with those of the control group, the levels of TNF-α and IL-6 in the serum and hippocampus homogenate were increased in rats after treatment with LPS. To evaluate the influence of DEX on TNF-α and IL-6 expression in rats, rats were pretreated with DEX before LPS stimulation. Our results indicated that DEX at concentration of 50 μg/kg significantly inhibited rats' TNF-α and IL-6 level induced by LPS, while 5 μg/kg show no anti-inflammatory effect in rats (Fig 2) (Table C, D, E and F in S1 File).

### Effect of DEX on NF-κB expression in rats

**Fig 3:**
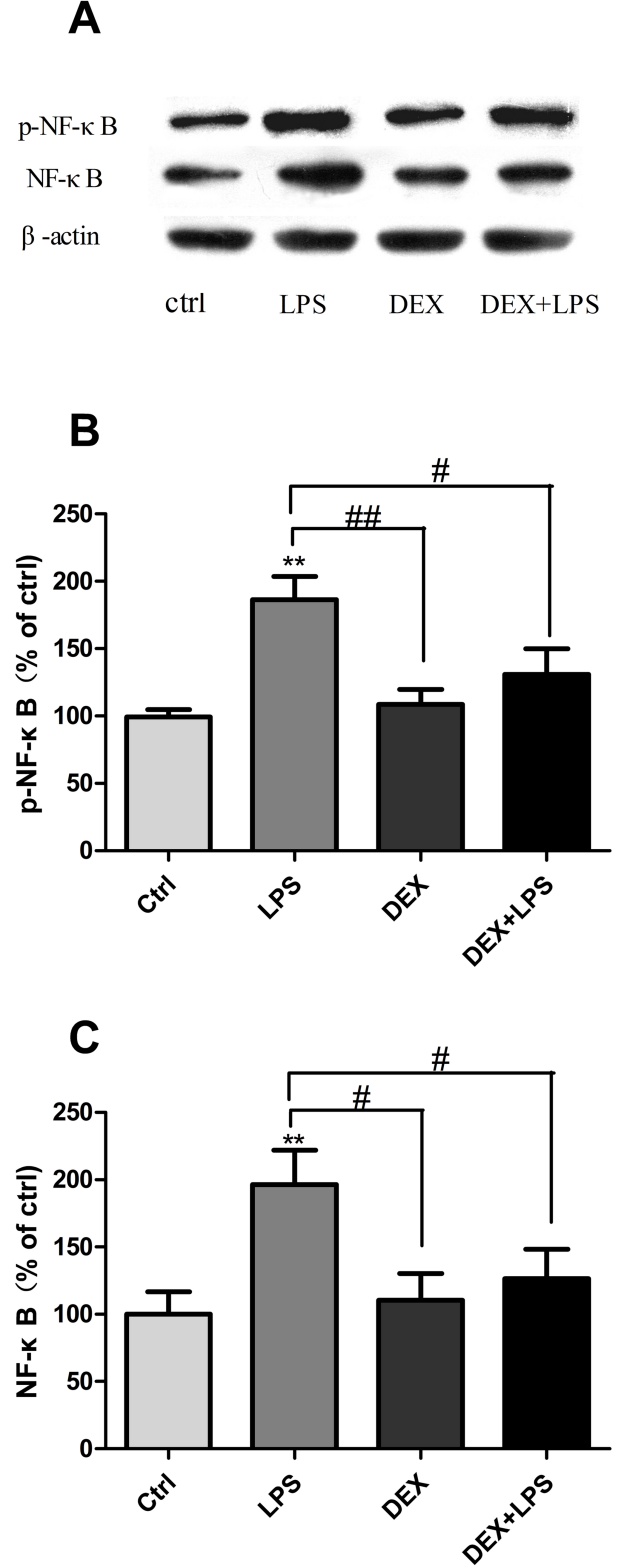
Rats pretreated with DEX showed a decrease in p-NF-κB and NF-κB expression. Western blotting showed that high dose of DEX reduced p-NF-κB and NF-κB protein levels in the hippocampus. (**P<0.01 versus control group; ^#^ P<0.05 versus LPS group).

Based on above results, we further test the mechanism of the anti-inflammatory effect of DEX. DEX at concentration of 50 μg/kg was used because of its anti-inflammatory effect. Rats were pretreated with DEX(50 μg/kg) before LPS stimulation, our results showed that DEX significantly decreased p-NF-κB and NF-κB expression induced by LPS in hippocampus (Fig 3) (Table G and H in S1 File).

### Effect of NF-κB inhibitor on TNF-α and IL-6 expression in rats

**Fig 4:**
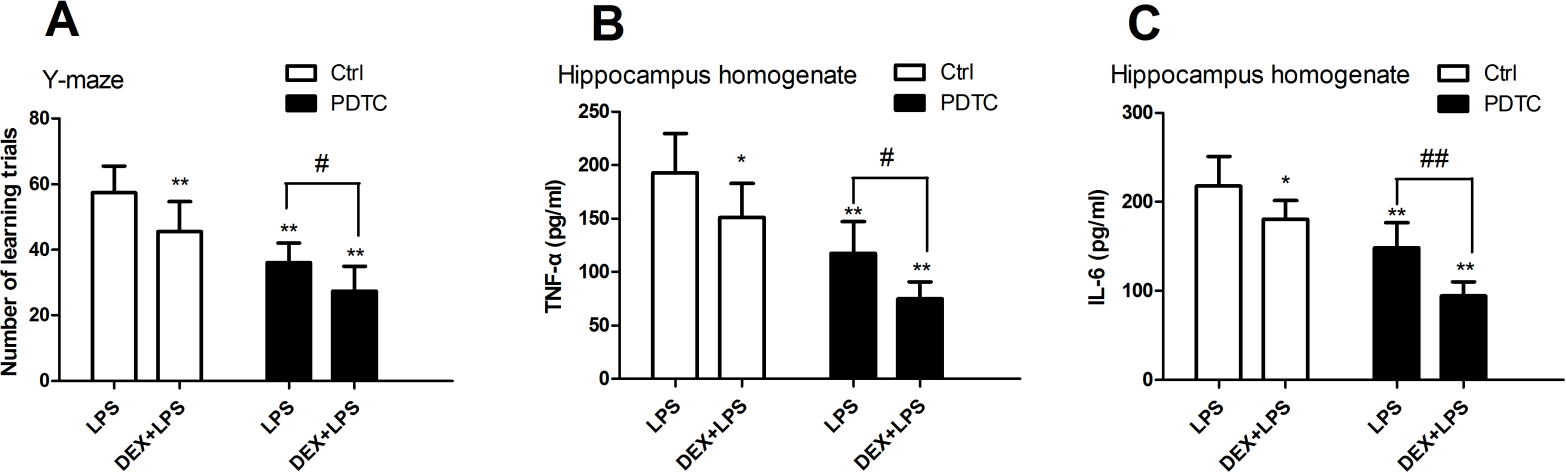
Pretreatment of PDTC and DEX in rats attenuated LPS-induced cognitive impairment and elevation of TNF-α and IL-6. Rats were treated with PDTC before DEX and LPS stimulation. Y-maze was used to estimate cognitive function of rats and expression of TNF-α and IL-6 was measured by ELISA. LPS decrease cognitive function and rats treated with LPS showed a significant attenuation of cognitive function. DEX and PDTC, each alone showed attenuation of LPS-induced cognitive impairment; in combination, they showed significant additive effects. Combined treatment with PDTC and DEX decreased LPS-induced TNF-α and IL-6 expression. (Mean ± SEM; **P < 0.01 compared with corresponding control group; #P < 0.05; ##P < 0.01 compared with LPS group).

To state the mechanism of anti-inflammatory effect of DEX, PDTC (NF-κB inhibitor, 50 mg/kg) was used by intraperitoneal injection before DEX and LPS stimulation. 24h after LPS treatment, rats were estimated with Y-maze, our results showed that the LPS decrease cognitive function as before (Fig 4A). Rats treated with LPS showed a significant attenuation of cognitive function. DEX and PDTC, each alone showed attenuation of LPS-induced cognitive impairment; in combination, PDTC and DEX showed significant additive effects(Table I in S1 File).

Rats treated with DEX showed a significant attenuation of TNF-α and IL-6 (19% and 17%) compared to LPS group. Rats treated with PDTC alone accumulated 39% and 36% less TNF-α and IL-6 than rats treated with LPS alone (Fig 4B and 4C). Rats treated with PDTC and DEX alongside LPS stimulation accumulated 61% and 57% less TNF-α and IL-6 than rats treated with LPS alone and significantly less than cells treated with either drug alone(Fig 4B and 4C) (Table J and K in S1 File). In combination with PDTC and DEX showed addictive effects. Therefore, DEX and PDTC can additively suppress LPS-induced TNF-α and IL-6 increase.

## Discussion

Our results showed that DEX inhibited TNF-α and IL-6 level induced by LPS in SD rats, and NF-κB might be involved in the anti-inflammatory effect of DEX. Accumulating evidence has indicated the release of pro-inflammatory cytokines plays an important role in inflammation in the CNS and cognitive impairment. LPS is a potent inflammatory cytokine stimulator. It is often used as a model to study sepsis, neuroinflammation and cognitive impairment. Several dosages of LPS were used in our study, and 100 μg/kg of LPS could stimulate moderate inflammation. Moreover, it has been reported that 100 μg/kg of LPS does not affect motor activity [15].

DEX has been widely used for its sedative effect. Recent studies demonstrated that besides the sedative effect, DEX also reduced post-operative delirium and POCD, which is correlated with post-operative cognitive impairment [16, 17].

Animal studies confirmed that post-operative cognitive impairment may be connected with neuroinflammation [18]. Selective inhibition of proinflammatory factors induced significantly reversed hippocampus-dependent cognitive deficits [19]. Neuroinflammation is also associated with melancholia and may account for multiple sclerosis (MS) and Alzheimer’s disease (AD) in older adults [20].

DEX has been proven to have neuroprotective effects in neuron impairment [21], and this effect may be partly via the activation of the PI3k/Akt/GSK3β pathway in the hippocampus of neonatal rats [22] and by up-regulating hypoxia-inducible factor-1α (HIF-1α) [23]. Some findings suggest it may be via inactivation of the TLR-4/NF-κB pathway in part [24], and inhibition of the NF-κB pathway may be a mechanism underlying the neuroprotective action of DEX against focal cerebral I/R injury [21]. However, the neuroprotective dosage of intravenous infusion of DEX is diverse in these studies. We found that 50 μg/kg DEX could exert neuroprotective effects in the hippocampus of adult rats. This result is probably due to the different stimulus position of transient ischemia and LPS in the brain. Our data also indicated that DEX at a higher dose (500 μg/kg) is harmful to the cognitive function of the rats. Therefore, we did not include this dosage in the following experiment.

Cytological experiments also verified that DEX is a potent suppressor of LPS-induced inflammation in astrocytes and microglia through α2A-adrenergic receptors [25, 26]. Our data demonstrated that LPS activated the NF-κB pathway and induced pro-inflammatory cytokines. This process could infer that LPS could bind toll-like receptor 4s (TLR-4) on the cell surface [27], activating NF-κB, leading to TNF-α and IL-6 mRNA synthesis. As the primary receptor for LPS, TLR4 was involved in pro-inflammatory cytokine releases induced by LPS in astrocytes [28]. This study also indicated that in combination with a specific NF-κB inhibitor (PDTC), DEX could enhance the attenuation of TNF-α and IL-6 in LPS-induced rats. These data are in-consistent with the study showing that DEX preconditioning suppresses retinal I/R injury and shows an effective anti-inflammatory effect by inhibiting TLR4/NF-κB expression [29].

Our study also suffers from several noteworthy limitations. The Y-maze was used to assess cognitive impairment in this study, and several other methods such as trace fear conditioning (TFC) should be applied as a supplement to confirm memory impairment. Another limitation is the concentrations of DEX. We found that 50 μg/kg could exert anti-inflammatory effects, while the clinical concentration is 0.5–10 μg/kg [30, 31], and the use of DEX at high concentrations (5–15 μg/kg) may be clinically practicable[32, 33]. Further studies are needed to confirm the optimal concentrations of DEX in animals.

In conclusion, our data demonstrated that DEX exerts a neuroprotective effect through NF-κB in part after LPS-induced cognitive impairment.

## Supporting information

S1 File Original data. Table A and B in S1 File: Y-MAZE of different groups in Fig 1. Table C, D, E and F in S1 File: ELISA results of TNF-α and IL-6 in serum and in hippocampus. Table G and H in S1 File: Western blot result of p-NF-κB and NF-κB in hippocampus. Table I, J and K in S1 File: Y-MAZE of different groups in Fig 4, ELISA results of TNF-α and IL-6 in hippocampus.
(DOC)
