# Supplementary material for: Dexmedetomidine inhibits inflammatory reaction in the hippocampus of septic rats by suppressing NF-κB pathway
Source: PLoS One. 2018 May 3;13(5):e0196897. doi: 10.1371/journal.pone.0196897 (PMC5933780; doi:10.1371/journal.pone.0196897)
Supplement: S1 File — Table A and B in S1 File: Y-MAZE of different groups in Fig 1. Table C, D, E and F in S1 File: ELISA results of TNF-α and IL-6 in serum and in hippocampus. Table G and H in S1 File: Western blot result of p-NF-κB and NF-κB in hippocampus. Table I, J and K in S1 File: Y-MAZE of different groups in Fig 4, ELISA results of TNF-α and IL-6 in hippocampus. (DOC) [file pone.0196897.s001.doc]

**Supporting Information 1. Original data**

Table A in S1 File: Y-MAZE

| Group (n=5) | Ctrl | LPS 10 μg/kg | LPS 100 μg/kg | LPS 1000 μg/kg | DEX 5 μg/kg | DEX 50 μg/kg | DEX 500 μg/kg |
| --- | --- | --- | --- | --- | --- | --- | --- |
| 1 | 16 | 15 | 45 | 65 | 24 | 27 | 38 |
| 2 | 23 | 31 | 30 | 52 | 17 | 15 | 39 |
| 3 | 15 | 29 | 33 | 47 | 35 | 35 | 42 |
| 4 | 21 | 12 | 42 | 58 | 22 | 22 | 47 |
| 5 | 29 | 32 | 48 | 55 | 16 | 29 | 56 |
| Mean ± SEM | 20.8 ± 2.5 | 23.8 ± 4.3 | 39.6 ± 3.5^**^ | 55.4 ± 3.0^**^ | 22.8 ± 3.4 | 25.6 ± 3.4 | 44.4 ± 3.3^**^ |

Table B in S1 File: Y-MAZE

| Group (n=10) | Ctrl | LPS 1000 μg/kg | DEX 5 μg/kg+LPS | DEX 50 μg/kg+LPS |
| --- | --- | --- | --- | --- |
| 1 | 19 | 55 | 45 | 32 |
| 2 | 14 | 60 | 66 | 23 |
| 3 | 25 | 43 | 48 | 30 |
| 4 | 20 | 57 | 63 | 41 |
| 5 | 22 | 64 | 56 | 36 |
| 6 | 19 | 66 | 40 | 34 |
| 7 | 17 | 50 | 70 | 28 |
| 8 | 25 | 56 | 37 | 24 |
| 9 | 13 | 44 | 36 | 26 |
| 10 | 27 | 65 | 44 | 28 |
| Mean ± SEM | 20.1 ± 1.5 | 56.0 ± 2.6^**^ | 50.5 ± 3.9^**^ | 30.2 ± 3.5^*##^ |

Table C in S1 File: ELISA results of TNF-α in serum

| Group (n=8) | Ctrl | LPS | DEX 5 μg/kg+LPS | DEX 50 μg/kg+LPS |
| --- | --- | --- | --- | --- |
| 1 | 36.7 | 143.6 | 103.5 | 73.7 |
| 2 | 41.0 | 141.6 | 136.3 | 63.5 |
| 3 | 33.5 | 101.2 | 169.8 | 111.2 |
| 4 | 40.2 | 120.3 | 116.7 | 82.9 |
| 5 | 41.1 | 181.5 | 141.2 | 45.6 |
| 6 | 33.6 | 152.6 | 132.5 | 62.1 |
| 7 | 34.4 | 121.0 | 122.7 | 88.6 |
| 8 | 38.9 | 120.0 | 121.2 | 95.6 |
| Mean ± SEM | 37.4 ± 1.2 | 135.2 ± 8.8^**^ | 130.5 ± 7.0^**^ | 77.9 ± 7.4^**##^ |

Table D in S1 File: ELISA results of TNF-α in hippocampus

| Group (n=8) | Ctrl | LPS | DEX 5 μg/kg+LPS | DEX 50 μg/kg+LPS |
| --- | --- | --- | --- | --- |
| 1 | 27.7 | 83.6 | 38.5 | 43.7 |
| 2 | 32.0 | 81.6 | 71.3 | 33.5 |
| 3 | 24.5 | 41.2 | 74.8 | 55.8 |
| 4 | 31.2 | 60.3 | 51.7 | 52.9 |
| 5 | 32.1 | 121.5 | 76.2 | 25.6 |
| 6 | 24.6 | 92.6 | 67.5 | 32.1 |
| 7 | 25.4 | 61.0 | 57.7 | 58.6 |
| 8 | 29.9 | 60.0 | 56.2 | 65.6 |
| Mean ± SEM | 28.4 ± 1.2 | 75.2 ± 8.8^**^ | 61.7 ± 4.6^**^ | 46.0 ± 5.1^*##^ |

Table E in S1 File: ELISA results of IL-6 in serum

| Group (n=8) | Ctrl | LPS | DEX 5 μg/kg+LPS | DEX 50 μg/kg+LPS |
| --- | --- | --- | --- | --- |
| 1 | 41.5 | 113.0 | 100.3 | 122.9 |
| 2 | 29.3 | 160.5 | 168.5 | 121.0 |
| 3 | 55.8 | 165.9 | 210.1 | 164.6 |
| 4 | 35.0 | 158.2 | 144.2 | 126.8 |
| 5 | 34.1 | 164.7 | 107.9 | 146.2 |
| 6 | 30.7 | 205.4 | 166.2 | 97.3 |
| 7 | 39.7 | 167.8 | 136.1 | 82.4 |
| 8 | 39.5 | 190.6 | 151.7 | 120.3 |
| Mean ± SEM | 38.2 ± 2.9 | 165.8 ± 9.5^**^ | 148.1 ± 12.4^**^ | 122.7 ± 9.1^**##^ |

Table F in S1 File: ELISA results of IL-6 in hippocampus

| Group (n=8) | Ctrl | LPS | DEX 5 μg/kg+LPS | DEX 50 μg/kg+LPS |
| --- | --- | --- | --- | --- |
| 1 | 30.3 | 80.0 | 115.9 | 97.4 |
| 2 | 18.5 | 127.3 | 143.6 | 96.8 |
| 3 | 44.8 | 132.4 | 185.3 | 139.2 |
| 4 | 24.1 | 125.6 | 119.0 | 101.9 |
| 5 | 23.2 | 131.2 | 82.1 | 121.3 |
| 6 | 19.5 | 172.4 | 141.2 | 72.3 |
| 7 | 28.8 | 134.5 | 111.6 | 57.7 |
| 8 | 28.3 | 157.1 | 126.0 | 95.6 |
| Mean ± SEM | 27.2 ± 2.9 | 132.6 ± 9.5^**^ | 128.1 ± 10.6^**^ | 97.8 ± 9.0^**#^ |

Table G in S1 File: Western blot results of p-NF-κB in hippocampus

| Group (n=4) | Ctrl | LPS | DEX | DEX + LPS |
| --- | --- | --- | --- | --- |
| 1 | 84.95 | 148.43 | 99.25 | 110.60 |
| 2 | 98.13 | 169.13 | 86.69 | 87.89 |
| 3 | 110.13 | 226.78 | 110.43 | 158.29 |
| 4 | 103.89 | 201.19 | 138.25 | 166.96 |
| Mean ± SEM | 99.3 ± 5.4 | 186.4 ± 17.3^**^ | 108.7 ± 11.0^##^ | 130.9 ± 19.0^#^ |

Table H in S1 File: Western blot results of NF-κB in hippocampus

| Group (n=4) | Ctrl | LPS | DEX | DEX + LPS |
| --- | --- | --- | --- | --- |
| 1 | 64.95 | 138.43 | 69.25 | 80.60 |
| 2 | 78.13 | 169.13 | 83.69 | 97.89 |
| 3 | 133.13 | 246.78 | 150.43 | 169.29 |
| 4 | 123.89 | 231.19 | 138.25 | 157.96 |
| Mean ± SEM | 100.0 ± 16.8 | 196.3 ± 25.6^**^ | 110.4 ± 20.0^#^ | 126.4 ± 21.9^#^ |

Table I in S1 File: Y-MAZE

| Group (n=10) | LPS | DEX + LPS | PDTC+LPS | DEX + PDTC +LPS |
| --- | --- | --- | --- | --- |
| 1 | 61 | 43 | 35 | 33 |
| 2 | 56 | 49 | 47 | 26 |
| 3 | 57 | 42 | 36 | 17 |
| 4 | 56 | 40 | 43 | 24 |
| 5 | 68 | 51 | 39 | 23 |
| 6 | 62 | 42 | 39 | 25 |
| 7 | 48 | 68 | 32 | 19 |
| 8 | 58 | 39 | 28 | 41 |
| 9 | 41 | 35 | 31 | 31 |
| 10 | 67 | 46 | 31 | 35 |
| Mean ± SEM | 57.4 ± 2.6 | 45.5 ± 2.9^**^ | 36.1 ± 1.9^**^ | 27.4 ± 2.4^*##^ |

Table J in S1 File: ELISA results of TNF-α in hippocampus

| Group (n=6) | LPS | DEX + LPS | PDTC+LPS | DEX + PDTC +LPS |
| --- | --- | --- | --- | --- |
| 1 | 234.3 | 220.6 | 117.0 | 79.7 |
| 2 | 209.5 | 112.9 | 153.3 | 61.2 |
| 3 | 203.6 | 140.1 | 69.4 | 62.6 |
| 4 | 183.8 | 120.0 | 102.1 | 99.3 |
| 5 | 200.2 | 180.7 | 142.3 | 85.0 |
| 6 | 125.4 | 161.7 | 120.8 | 61.4 |
| Mean ± SEM | 192.8 ± 15.1 | 151.0 ± 13.0^*^ | 117.5 ± 12.2^**^ | 74.9 ± 6.4^**#^ |

Table K in S1 File: ELISA results of IL-6 in hippocampus

| Group (n=6) | LPS | DEX + LPS | PDTC+LPS | DEX + PDTC +LPS |
| --- | --- | --- | --- | --- |
| 1 | 226.9 | 179.8 | 109.8 | 81.2 |
| 2 | 194.4 | 210.9 | 156.0 | 102.6 |
| 3 | 217.3 | 167.9 | 170.0 | 92.5 |
| 4 | 267.2 | 155.0 | 167.2 | 114.9 |
| 5 | 169.9 | 168.3 | 171.4 | 103.4 |
| 6 | 230.0 | 199.6 | 115.8 | 71.4 |
| Mean ± SEM | 217.6 ± 13.6 | 180.2 ± 8.7^*^ | 148.4 ± 11.5^**^ | 94.3 ± 6.5^**##^ |
